# Analysis of biofilm production by clinical isolates of *Pseudomonas aeruginosa* from patients with ventilator-associated pneumonia

**DOI:** 10.5935/0103-507X.20170039

**Published:** 2017

**Authors:** Jailton Lobo da Costa Lima, Lilian Rodrigues Alves, Jussyêgles Niedja Pereira da Paz, Marcelle Aquino Rabelo, Maria Amélia Vieira Maciel, Marcia Maria Camargo de Morais

**Affiliations:** 1 Postgraduate Program in Tropical Medicine, Health Sciences Center, Universidade Federal de Pernambuco - Recife (PE), Brazil.; 2 Postgraduate Program in Applied Cellular and Molecular Biology, Institute of Biological Sciences, Universidade de Pernambuco - Recife (PE), Brazil.

**Keywords:** Pseudomonas aeruginosa, Biofilms, Respiration, artificial/adverse effects, Pneumonia, ventilator-associated

## Abstract

**Objective:**

To phenotypically evaluate biofilm production by *Pseudomonas aeruginosa* clinically isolated from patients with ventilator-associated pneumonia.

**Methods:**

Twenty clinical isolates of *P. aeruginosa* were analyzed, 19 of which were from clinical samples of tracheal aspirate, and one was from a bronchoalveolar lavage sample. The evaluation of the capacity of *P. aeruginosa* to produce biofilm was verified using two techniques, one qualitative and the other quantitative.

**Results:**

The qualitative technique showed that only 15% of the isolates were considered biofilm producers, while the quantitative technique showed that 75% of the isolates were biofilm producers. The biofilm isolates presented the following susceptibility profile: 53.3% were multidrug-resistant, and 46.7% were multidrug-sensitive.

**Conclusion:**

The quantitative technique was more effective than the qualitative technique for the detection of biofilm production. For the bacterial population analyzed, biofilm production was independent of the susceptibility profile of the bacteria, demonstrating that the therapeutic failure could be related to biofilm production, as it prevented the destruction of the bacteria present in this structure, causing complications of pneumonia associated with mechanical ventilation, including extrapulmonary infections, and making it difficult to treat the infection.

## Abstract (pt)

**Objetivo:**

Avaliar fenotipicamente a produção de biofilme por isolados clínicos de *Pseudomonas aeruginosa* de pacientes com pneumonia associada à ventilação mecânica.

**Métodos:**

Foram analisados 20 isolados clínicos de *P. aeruginosa*, sendo 19 provenientes de amostras clínicas de aspirado traqueal e uma de lavado broncoalveolar. A avaliação da capacidade de *P. aeruginosa* em produzir biofilme foi verificada por duas técnicas, sendo uma qualitativa e outra quantitativa.

**Resultados:**

A técnica qualitativa mostrou que apenas 15% dos isolados foram considerados produtores de biofilme, enquanto que a quantitativa demonstrou que 75% dos isolados foram produtores de biofilme. Os isolados produtores de biofilme apresentaram o seguinte perfil de suscetibilidade: 53,3% eram multidroga-resistentes e 46,7% eram multidroga-sensíveis.

**Conclusão:**

A técnica quantitativa foi mais eficaz para detecção da produção de biofilme em comparação com a qualitativa. Para a população bacteriana analisada, a produção de biofilme independeu do perfil de suscetibilidade das bactérias, demonstrando que a falha terapêutica pode estar relacionada com a produção de biofilme, por impedir a destruição das bactérias presentes nesta estrutura, ocasionando complicações da pneumonia associada à ventilação mecânica, incluindo infecções extrapulmonares, e dificultando o tratamento da infecção.

## INTRODUCTION

Patients with respiratory and metabolic weakness use mechanical ventilation (MV), a method of artificial ventilation that ensures the maintenance of gas exchange essential for the body and is considered a therapeutic support commonly used in intensive care units (ICUs). However, it exposes patients to the risk of acquiring ventilator-associated pneumonia (VAP).^(1)^ It is suggested that the tracheal tube acts as a trigger for VAP by the formation of biofilm on its surface, favoring the pathogenesis of the infection.^(2)^ Moreover, the microbial interaction within the biofilm may contribute to the pathogenesis of VAP and have an impact on antimicrobial therapy, increasing the morbidity and mortality rates associated with this infection.^(3)^ To reduce biofilm formation in the endotracheal tube during MV and, consequently, to reduce the frequency of VAP, decontamination of the oral microbiota and reduction of dental plaques have been used since both are potential sources for the onset of VAP.^(4,5)^

The diagnosis of pneumonia is complex. The three main components for the detection of VAP according to the current criteria are chest radiography (mandatory), signs and symptoms (mandatory) and laboratory tests (optional). There is still no gold standard for the diagnosis of this infection, and most of the definitions used do not have sufficient sensitivity or specificity to establish this diagnosis.^(6)^ Microbiological data are used in an attempt to refine the diagnostic accuracy due to the low specificity of the clinical criteria alone.^(7)^

Often, the pathogens that cause early-onset VAP (diagnosed up to the fourth day after starting MV use) are of community origin. These pathogens include *Streptococcus pneumoniae, Haemophilus influenzae, Moraxella catarrhalis*, methicillin-resistant *Staphylococcus aureus* (MRSA) or enterobacteria susceptible to antimicrobials.^(8,9)^ Late-onset VAPs (diagnosed from the fifth day after the start of MV use) are caused by opportunistic pathogens, such as *Pseudomonas aeruginosa, Acinetobacter spp.* and other resistant opportunistic *Gram-*negative bacteria, in addition to MRSA.^(9)^

*P. aeruginosa* is currently considered the main agent of VAP in the ICU, causing an average of approximately 50% of VAP cases.^(10,11)^ The infections caused by this microorganism occur predominantly in critical and immunocompromised patients and are associated with the increasing morbidity and mortality of patients in these units, in addition to the increase in cases of *P. aeruginosa* resistance to antimicrobials.^(12,13)^ Although VAP is the main infection related to health care caused by this microorganism, its involvement in the etiology of other infections, such as infections of the urinary tract, surgical sites and, mainly, sepsis, is also important.^(14,15)^

The biofilm production by the microorganisms that cause VAP makes the antimicrobial therapy of this infection even more difficult since the biofilm acts as a barrier, reducing the penetration of these drugs and, consequently, preventing them from exercising their actions, along with hindering the recognition of the microorganisms by the host immune system.^(2)^ In view of the above, the objective of this work was to phenotypically evaluate the biofilm production by clinical isolates of *P. aeruginosa* from patients with VAP.

## METHODS

A total of 20 clinical isolates of *P. aeruginosa* stored in the bacterial collection of the Laboratory of Bacteriology and Molecular Biology of the *Universidade Federal de Pernambuco* (UFPE) were analyzed; 19 were from clinical samples of tracheal aspirates, and 1 was from a bronchoalveolar lavage sample. These isolates were collected during the period from November 2012 to November 2013 and were stored frozen at -20ºC. The confirmation of the diagnosis of VAP in the patients was based on clinical and microbiological criteria of the hospital, obtained from the analysis of the medical records of the patients, and the study was approved by the Research Ethics Committee of UFPE, registered at CEP/CCS/UFPE under number 009/11.

The isolates were previously identified, and the susceptibility analysis was performed using an automated system (Phoenix - BD^®)^, where isolates with resistance to at least three classes of drugs from a variety of antimicrobial classes (mainly aminoglycosides, penicillins, cephalosporins, carbapenems and fluoroquinolones) were considered multidrug-resistant (MDR), and isolates that showed resistance to two or fewer classes of antimicrobials were considered multidrug-sensitive (MDS).^(16)^ Subsequently, the isolates were sent to the Laboratory of Bacteriology and Molecular Biology, where they were kept frozen in glycerol at -20ºC in the laboratory's bacterial collection. These bacteria were reactivated in test tubes containing brain heart infusion (BHI) broth, incubated for 48 hours in an oven at 37ºC, seeded in cetrimide agar and placed in an oven at 37ºC for 24 hours for analysis.

### Phenotypic characterization of biofilm production

#### Congo red agar test

The evaluation of the capacity of *P. aeruginosa* to produce a capsule as a presumptive test for biofilm formation was performed using the Congo red agar method following the protocol described in 1989.^(17)^ In this test, Congo red dye was used as a pH indicator, showing black coloration at pH ranges between 3.0 and 5.2. Plates with the Congo red agar medium were seeded and incubated in an aerobic environment for 24 to 48 hours at 37ºC. After this period, colonies that were dark red or blackish in color, with dry or crystalline consistency, were considered biofilm producers; red colonies with a smooth and darkened appearance in the center were considered biofilm non-producers. Colonies of *Klebsiella pneumoniae* and *Citrobacter sp*. from the bacterial collection of the Laboratory of Bacteriology and Molecular Biology of UFPE were used as positive and negative controls, respectively. The reference strain *P. aeruginosa* (PA01) was also used as a positive control of the test because this strain has been characterized as a biofilm producer.

### Biofilm production test

The biofilm quantification assay was performed using a previously described technique,^(18)^ with modifications, with BHI broth and the addition of sucrose at a concentration of 50 g/L. Isolates of *P. aeruginosa* were cultured in BHI broth for 24 hours at 37ºC.

For microtitration, 200 µL of the bacterial suspensions were applied in triplicate on polystyrene plates containing 96 flat-bottom wells; BHI broth without bacterial inoculum was used as the negative control, and *P. aeruginosa* strain PA01 was used as the positive control since this strain is recommended as a positive control for biofilm assays. The plates were then incubated at 37ºC for 24 hours. The bacterial suspensions were then removed, and each well was washed three times with 250 µL of sterile saline solution (0.9% NaCl). Subsequently, fixation with 200 µL of methanol pa was performed for 15 minutes. The methanol was removed, the plates were left at room temperature to dry and they were stained with 200 µL of crystal violet solution for 5 minutes. The plates were then washed with running water and dried at room temperature. After this process, absorbance readings were taken in an ELISA reader (BioRad, model 550) at wavelength of 570 nm, and the samples were classified according to Stepanovic et al.^(18)^ The value of the optical densities for each isolate (OD_i_) was obtained by averaging the three wells, and this value was compared to the optical density of the negative control (ODc). The isolates were classified into four categories, according to the mean optical densities (OD) in relation to the ODc results. The categories were based on the following criteria: non-adherent if OD_i_ ≤ ODc; weakly adherent (+) if ODc < OD_i_ ≤ 2 x ODc; moderately adherent (++) if 2 x ODc < OD_i_ ≤ 4 x ODc; or strongly adherent (+++) if 4x ODc < OD_i_.

## RESULTS

### Congo red agar test

The Congo red agar test showed low positivity in the presumptive detection of biofilm production in 15% of the analyzed *P. aeruginosa* isolates, and the three isolates were MDS.

### Biofilm production test

Biofilm quantification analyses showed that 75% of the isolates were biofilm producers, indicating that this technique was more efficient than Congo red agar for the detection of biofilm production. The clinical isolates of this study had the following results for the categories of biofilm production: 25% were non-adherent, 40% were weakly adherent, 25% were moderately adherent, and 10% were strongly adherent.

Of the three isolates considered to be biofilm producers by the Congo red agar technique, two were also biofilm producers by the quantification technique. Table 1 shows the relationship between the adhesion profile found in the *P. aeruginosa* isolates analyzed and the susceptibility profile. The results from this study are summarized in table 2, including the type of sample analyzed, the susceptibility profiles of the clinical isolates and the results obtained in the biofilm detection tests.

**Table 1:** Adhesion profiles *versus* susceptibility of the clinical isolates

Adhesion profile	Multidrug-resistant	Multidrug-sensitive
Non-adherent	2	3
Weakly adherent	4	4
Moderately adherent	4	1
Strongly adherent	0	2
Total	10	10

**Table 2:** Susceptibility profiles of clinical isolates versus biofilm production

Isolate	Sample	Adhesion profile	Multidrug-resistant	Congo red agar	Biofilm quantification
P3AM	Tracheal secretion	Imipenem	No	Positive	Non-adherent
P8AM	Tracheal secretion	Amikacin, gentamicin, tobramycin, aztreonam, ceftazidime, cefepime, ciprofloxacin, levofloxacin, imipenem and meropenem	Yes	Negative	Weakly adherent
P9AM	Bronchoalveolar lavage	No resistance	No	Positive	Weakly adherent
P13AM	Tracheal secretion	Aztreonam, imipenem and meropenem	No	Positive	Weakly adherent
P22AM	Tracheal secretion	Ciprofloxacin and levofloxacin	No	Negative	Non-adherent
P23AM	Tracheal secretion	No resistance	No	Negative	Weakly adherent
P24AM	Tracheal secretion	No resistance	No	Negative	Strongly adherent
P25AM	Tracheal secretion	Gentamicin, aztreonam, ciprofloxacin, levofloxacin and piperacillin-tazobactam	Yes	Negative	Moderately adherent
P28AM	Tracheal secretion	Amikacin, gentamicin, tobramycin, aztreonam, ceftazidime, cefepime, ciprofloxacin, levofloxacin, imipenem, meropenem and piperacillin-tazobactam	Yes	Negative	Moderately adherent
P29AM	Tracheal secretion	No resistance	No	Negative	Strongly adherent
P32AM	Tracheal secretion	Gentamicin, tobramycin, aztreonam, ceftazidime, cefepime, ciprofloxacin, levofloxacin, imipenem, meropenem and piperacillin-tazobactam	Yes	Negative	Moderately adherent
P30HC	Tracheal secretion	Amikacin, gentamicin, tobramycin, aztreonam, ceftazidime, cefepime, ciprofloxacin, levofloxacin, meropenem and piperacillin-tazobactam	Yes	Negative	Non-adherent
P35HC	Tracheal secretion	Aztreonam, ceftazidime, cefepime, ciprofloxacin, imipenem and meropenem	Yes	Negative	Weakly adherent
P41HC	Tracheal secretion	Aztreonam, ceftazidime, cefepime, ciprofloxacin, levofloxacin, meropenem and piperacillin-tazobactam	Yes	Negative	Weakly adherent
P61HC	Tracheal secretion	Amikacin and ciprofloxacin	No	Negative	Moderately adherent
P73HC	Tracheal secretion	Amikacin, aztreonam, ceftazidime, cefepime, ciprofloxacin, levofloxacin, meropenem and piperacillin-tazobactam	Yes	Negative	Non-adherent
P123HC	Tracheal secretion	Amikacin, gentamicin, tobramycin, aztreonam, ceftazidime, cefepime, ciprofloxacin, imipenem and meropenem	Yes	Negative	Moderately adherent
P125HC	Tracheal secretion	Ceftazidime	No	Negative	Non-adherent
P129HC	Tracheal secretion	Piperacillin-tazobactam	No	Negative	Weakly adherent
P131HC	Tracheal secretion	Amikacin, gentamicin, tobramycin, aztreonam, ceftazidime, cefepime, ciprofloxacin, levofloxacin, imipenem, meropenem and piperacillin-tazobactam	Yes	Negative	Weakly adherent

## DISCUSSION

Studies evaluating the effectiveness of the Congo red agar test for *P. aeruginosa* are scarce. One study,^(19)^ analyzed the biofilm formation using the Congo red agar technique in strains of *S. aureus* (ATCC 29213), *Staphylococcus epidermidis* (clinical sample) and *P. aeruginosa* (ATCC 27853), while another study,^(20)^ observed the capacities of *S. aureus* (ATCC 25923) and *P. aeruginosa* (ATCC 27853) strains to produce biofilm by this technique. In both studies, all strains analyzed were considered biofilm producers. Another study,^(21)^ evaluated biofilm formation in 30 clinical isolates of *P. aeruginosa* using this method and identified 27 biofilm producers.

Although the Congo red agar test is widely used in biofilm studies, mainly with *Staphylococcus* spp., the specific mechanism of the response involved in this method is unknown. However, some data indicate that a positive reaction, evidenced by the darkening of a biofilm-producing colony, would result from the polysaccharide constitution of the extracellular matrix of the biofilm, whose production is intensified by the nutritional supplement of the medium.^(17)^

In studies performed using this method, an association between the polysaccharide production and the positive reaction in the Congo red agar test was detected in a biofilm-producing *S. epidermidis* species that had *Operon ica* genes. This association was also observed in other biofilm-producing species of the genus *Staphylococcus* that have genes homologous to the *Operon ica* of *S. epidermidis* (*S. aureus, Staphylococcus caprae, Staphylococcus lugdunensis and Staphylococcus haemolyticus*).^(22,23)^ In addition, positivity was observed in this test for other biofilm-producing bacterial genera/species that had genes orthologous to *Operon ica,* such as *Actinobacillus pleuropneumoniae*,^(24)^ *Aggregatibacter actinomycetemcomitans*,^(25)^ *Bordetella*,^(26)^ *Escherichia coli*^(27)^ and *Yersinia pestis*.^(28)^

In this study, it was not possible to observe the relationship between the production of the extracellular matrix of the *P. aeruginosa* biofilm, predominantly consisting of polysaccharide (alginate), and the Congo red agar test positivity since even the *P. aeruginosa* PA01 strain, which is a positive control for biofilm production tests, was not positive in this test, demonstrating that this test is not effective for the presumptive detection of biofilm formation for this bacterial species. The lack of positivity in the Congo red test may be related to the deficiency of the gene *pel*, which is responsible for the production of the glucose-rich extracellular matrix and is capable of binding to Congo red and generating the reaction that modifies the coloration of the biofilm-producing colonies. No extracellular matrix is produced by the mutant *P. aeruginosa* isolates, which do not express the *pel* gene, thus causing a lack of positivity in the Congo red agar test.^(29)^

The biofilm quantification test was effective in the detection of biofilm production by clinical isolates from patients with VAP and was also able to verify biofilm production by the *P. aeruginosa* PA01 strain, which was used as a positive control in the test. Similar data have been recorded in the literature,^(30)^ showing a biofilm production of 68% (50/74) in clinical isolates of *P. aeruginosa*, which were distributed in the following categories: 96% weakly adherent and 4% moderately adherent.

In this study, isolates classified as biofilm producers had the following susceptibility profile: 53.3% were MDR, and 46.7% were MDS. Due to the small sample size, a statistical analysis was not performed to evaluate the difference between the results of the MDR and MDS isolates, although there was greater biofilm production in MDR isolates, corroborating previous data^(30)^ in which metallo-β-lactamase (MβL)-producing *P. aeruginosa* isolates produced biofilm. These results are also similar to others in the literature,^(31)^ which showed biofilm production of 93.4% (85/91), with 60% being weakly adherent, 25.9% moderately adherent and 14.1% strongly adherent.

The data obtained in this study showed that for the bacterial population studied, the biofilm production was independent of the susceptibility profile of the bacteria. Biofilm production may be related to the failure of empirical therapy, as the biofilm reduces the penetration of antimicrobials, preventing them from eliminating the bacteria present in the biofilm and causing VAP complications in patients, including extrapulmonary infections, making it difficult to treat the infection. This finding is very important because within the same hospital, the empirical regimens for the treatment of VAP can differ according to the circulating etiological agents and their susceptibility profiles. We also suggest that the therapeutic protocols of VAP should be systematically reviewed to determine treatment success and to minimize the selective pressure of resistant microorganisms.^(32)^

Biofilm formation in the endotracheal tubes of patients with VAP prolongs this condition and spreads the infection to other regions of the proximal respiratory tract, resulting in increased use of antimicrobials to control the infection. However, in most cases, this therapy is not successful due to the reduction of the penetration of the antimicrobial agents caused by the biofilm formation. When the degree of adhesion of the biofilm is higher, the penetration of the antimicrobial into its structure is reduced, leading to selective pressure in the cells present and resulting in the increase of the resistance of this bacterium by this and/or other mechanisms of resistance.^(33)^

In this study, the qualitative technique revealed that only 15% of the isolates were considered biofilm producers, while the biofilm quantitative technique revealed that 75% of the isolates were biofilm producers, indicating that the quantitative technique was more efficient than the qualitative technique for the detection of biofilm production. There was also high biofilm production by the evaluated clinical isolates of *P. aeruginosa*.

## CONCLUSION

Our study demonstrated the greater detection of biofilm production by clinical isolates of *P. aeruginosa* from patients with ventilator-associated pneumonia using the quantitative technique, which was more effective than the qualitative technique. Moreover, for the microorganisms evaluated in this study, biofilm production was independent of the susceptibility profiles of the bacteria. Studies on biofilm production by *P. aeruginosa* are still scarce in Brazil, and there are no reports on biofilm production by this bacterium related to ventilator-associated pneumonia in the country. Further studies with a larger number of clinical isolates of *P. aeruginosa* from patients with ventilator-associated pneumonia are needed to elucidate the dynamics of this infection and the formation of biofilm by this microorganism to improve patient quality of life.
